# What Is the Most Appropriate Source for Hematopoietic Stem Cell Transplantation? Peripheral Stem Cell/Bone Marrow/Cord Blood

**DOI:** 10.1155/2012/834040

**Published:** 2012-09-27

**Authors:** Itır Sirinoglu Demiriz, Emre Tekgunduz, Fevzi Altuntas

**Affiliations:** Hematology Clinic and Hematopotic Stem Cell Transplantation Unit, Ankara Oncology Training and Research Hospital (AOH), Yenimahalle, Demetevler, 06200 Ankara, Turkey

## Abstract

The introduction of peripheral stem cell (PSC) and cord blood (CB) as an alternative to bone marrow (BM) recently has caused important changes on hematopoietic stem cell transplantation (HSCT) practice. According to the CIBMTR data, there has been a significant decrease in the use of bone marrow and increase in the use of PSC and CB as the stem cell source for HSCT performed during 1997–2006 period for patients under the age of 20. On the other hand, the stem cell source in 70% of the HSCT procedures performed for patients over the age of 20 was PSC and the second most preferred stem cell source was bone marrow. CB usage is very limited for the adult population. Primary disease, stage, age, time and urgency of transplantation, HLA match between the patient and the donor, stem cell quantity, and the experience of the transplantation center are some of the associated factors for the selection of the appropriate stem cell source. Unfortunately, there is no prospective randomized study aimed to facilitate the selection of the correct source between CB, PSC, and BM. In this paper, we would like to emphasize the data on stem cell selection in light of the current knowledge for patient populations according to their age and primary disease.

## 1. Trials Comparing Bone Marrow and Peripheral Stem Cell

One of the main reasons for preferring PSC worldwide is the important advantages provided by this method to the donor. These advantages are avoidance of anesthesia, lack of the need for hospitalization or blood transfusion, and very low serious adverse event risk. The largest trial to date comparing these different stem cell sources in HLA matched sibling donor setting was the meta-analysis of IBMTR/EBMT including 536 and 288 patients, who received BM and PSC, respectively [1]. In this trial, a faster neutrophil and platelet engraftment were observed in PSC arm. However, there was no statistically significant difference for relapse and grade II–IV acute graft-versus-host disease (aGvHD) between groups. After 1 year of followup, chronic GVHD frequency was significantly higher in the PSC (65%) arm compared to BM (53%) arm.

Between 1998 and 2002, BM and PSC as a stem cell source were compared in 8 randomized trials [2–9]. Almost all of the patients included were diagnosed as leukemia. Number of patients included, remission status, conditioning regimen, GvHD prophylaxis, stem cell, and T-cell numbers were significantly different in these studies. Combined results suggest faster neutrophil and platelet engraftment with PSC compared to BM. One of the trials revealed similar grade II–IV aGVHD incidence. The largest randomized EBMT study has shown that the use of PSC significantly increased both the frequency of grade II–IV aGvHD (52%–39%; *P*: 0.013) and cGvHD (67%–54%; *P*: 0.0066) [5]. In EBMT trial omission of methotrexate on day 11 for aGVHD prophylaxis was suggested to be responsible for the increased aGVHD incidence; however, another meta-analysis was not able to verify this hypothesis [10]. Two out of four large-scale randomized trials showed increased chronic GVHD frequency (%22 and 13%) in patients treated with PSC [3, 5]. Other trials did not report statistically significant increase in chronic GVHD although an insignificant trend for increased cGvHD was observed [2, 4]. The long-term results of French [11] and EBMT [12] trials indicated a higher frequency of chronic GVHD in the PSC compared to BM group; however, the short- and the long-term follow-up results of the North American [13] trial did not support these findings.

Graft versus tumor effect is mostly associated with the T lymphocytes. As the number of T lymphocytes is higher in PSC product compared to BM, we should expect a lower relapse rate in HSCT using PSC as stem cell source, whereas randomized trials do not report any decrease in relapse risk by using PSC.

Retrospective evaluation reveals lower transplant related mortality (TRM) in HSCT with PSC. IBMTR/EBMT results showed significant decrease in TRM with PSC in the advanced stage leukemia patients undergoing HSCT [1]. Randomized trials did not report statistically significant difference for TRM between PSC and BM. However, we cannot comment on the effect of disease subgroups, stages, and stem cell source which may have significant impact on TRM.

The most distinctive end point of HSCT is the overall survival. There are three big randomized trials reporting different results for this end point. EBMT [5] trial did not find any difference in terms of survival between BM and PSC; but another trial performed in the USA has reported a trend for increased 2-year overall survival (*P*: 0.06) for PBC [2]. After 30 months of followup, Canadians reported a %8 (*P*: 0.04) survival advantage in the PSC arm of the trial [14]. USA and Canadian trials in common indicated a survival advantage gained with PSC in advanced disease stages.

The meta-analysis of nine trials including a total of 1111 patients provides us very important data on this topic [15]. The meta-analysis confirms that the selection of PSC decreases the duration of neutrophil and platelet engraftment increases the frequency of grade III–IV aGVHD and chronic GVHD compared to BM. PSC use decreases the 3-year relapse rates in both early stage (%16–%20; *P*: 0.04) and advanced stage (%33–%51; *P*: 0.02) diseases compared to BM. On the other hand, PSC only increases the 3-year overall survival (%46–%31; *P*: 0.01) in the advanced stage disease group. It has been hard to generalize the results of these trials included in this meta-analysis because the majority of the patients had early stage disease such as chronic phase CML (75%) and AML in first complete remission.

Trials comparing PSC and BM as the stem cell source included almost only leukemia patients; on the other hand, very few patients diagnosed with lymphoma and myeloma have been included. Data on the stem cell source in HSCT setting for benign hematological diseases are not sufficient. EBMT has evaluated 692 severe aplastic anemia patients; over the age 20, there was no significant difference between PSC and BM in terms of cGVHD and mortality. In younger patients, cGVHD and mortality rates were higher (%27–%12) in the PSC group [16]. 5-year survival was increased %12 (%85–%73) when BM was preferred as the stem cell source in the subgroup of patients with the age ≤ 20. In conclusion, these data suggests that the type of the disease and the age of the patient play a role in deciding the optimal source of stem cell (Table 1).

Consequently, the randomized trials including adult patients point out that PSC increases chronic GVHD, provides overall survival advantage for advanced stage leukemia patients, but does not significantly have an effect on survival of early stage leukemia patients. On the other hand, in none of these trials, the followup duration has exceeded 3 years and the long-term results are still not known.

The joint IBMTR/EBMT study retrospectively evaluated a large patient population for the long-term results of PSC and BM as a stem cell source [17]. Between 1995-1996 patients over the age of 20 and with different stages of AML, ALL, and CML who underwent HSCT from PSC (*n*: 288) and BM (*n*: 462) has been analyzed. Follow-up data on 413 surviving patients (BM: 272; PSC: 141) has been evaluated and a median of six years followup results has been reported. Chronic GVHD incidence was 61% in PSC group and 45% in BM group. PSC has decreased TRM in the advanced stage AML and ALL subgroup but did not increase TRM in the chronic phase CML cases. For acute leukemia patients in first complete remission, there was no significant difference on survival according to stem cell source. But in patients, who achieved second complete remission, there was a trend for increased survival with use of PBC compared to BM (%49–%42). The effectiveness of stem cell source changes according to the stage in chronic leukemia patients. In chronic phase patients, BM provided 6-year survival advantage (%64–%43), but in the accelerated phase disease PSC seems to be superior in terms of survival (%33–%25).

Another trial from IBMTR included 773 (BM: 630; PSC: 143) acute leukemia patients 8–20 of age, who underwent HSCT from HLA match sibling donor between 1995–2000. The followup period was 4 years. Chronic GVHD frequency was 33% in PSC group and 19% in BM group. PSC has increased overall survival by %10 (%58–%48). There was no significant difference between stem cell sources in aGVHD and relapse rates.

CIMBTR data was analyzed for stem cell source of unrelated donors. This trial has evaluated 911 (PSC: 331; BM: 586) patients aged 18–60 years who were diagnosed as AML, ALL, CML, and MDS between 2000 and 2003. The frequency of aGVHD on posttransplantation day 100 (%58–%45) and chronic GVHD (%56–%42) were increased in significantly higher in the PSC group. Three years TRM (%45–%44) and the overall survival (%32–%30) showed no significant difference between PSC and BM groups.

## 2. The Role of Cord Blood

In theory, all of the patients who are candidates for HSCT but do not have a matched sibling donor but can provide adequate cord blood are candidates for HSCT with CB. HSCT with CB can be performed with 4/6 or 3/6 match, this is why %99 of all patients belonging to all ethnic groups can find acceptable CB units [17]. Therefore, CB is a very important stem cell source alternate but in adult patients stem cell number may be inadequate and there are disadvantages such as longer duration for engraftment and accompanying infections. HSCT with CB has been increased in last two decades; for adult patients, double CB transplantations have been performed successfully but there is no prospective randomized trial head to head comparing CB, PSC, and BM as a stem cell source.

IBMTR [18] and Eurocord [19] trials have retrospectively evaluated the CB use from sibling donors for pediatric patients; under the age of 15 and 1 year survival has been reported to be above 60%. Eurocord trial compared CB (*n*: 113) with BM (*n*: 2052). CB recipients were 3 years younger (*P* < 0.001), 9 kilograms lighter (*P* < 0.001), and were treated with lower doses of methotrexate (%28–%65; *P* < 0.001) for aGVHD prophylaxis. The median cell number was 4.7 × 10^7^ nucleated cell/kg in CB recipients. Neutrophil and platelet engraftment ratios were lower with CB compared to BM. Grade II–IV aGVHD (%14–%24; *P* = 0.02) and chronic GVHD (%6–%15; *P* = 0.02) frequency were significantly lower in patients who received CB. There was no difference between groups in terms of 3 years overall survival (CB: %64; BM: %66). These data suggests that CB from HLA matched sibling has similar outcomes as BM from HLA match sister/brother for the pediatric group.

Unrelated CB transplantation data is mostly based on retrospective analysis. Two of three New York Blood Center studies are conducted in 0–11 years old children who were diagnosed with a hematological malignancy and 87% of all grafts were one or two antigen mismatched. The most important factor for the neutrophil engraftment in this series of 861 cases has been found to be the infused cell number. Neutrophil engraftment duration was median of 5 days earlier (*P* = 0.0027) in transplantations with HLA full matched compared to the HLA mismatched CB transplants [20]. HLA mismatch increases the risk of severe GVHD. Grade III–IV aGVHD rate was 8% with HLA A, B, DRB1 matched transplantations, but in mismatched cases it has been increased to 28% (*P* = 0.006). Multivariate analysis revealed that the most important markers for relapse were the stage of the disease and GVHD. The 3-year survival rates are predicted as 27% and 47% in hematological malignancies and genetic diseases, respectively.

In adult patients, CIBMTR/EBMT has retrospectively evaluated a total of 1525 patients who underwent unrelated HSCT for acute leukemia between 2002 and 2006 and randomized them into 3 different groups (CB: 165; BM: 472; PSC: 888) [21]. Disease-free survival ratios were similar between 8/8 and 7/8 HLA matched BM, PSC cases, and CB recipients. Considering that the 70% of CB group received two antigen mismatched transplants, this success of CB is remarkable. On the other hand, TRM was higher in the CB group when compared with 8/8 HLA matched PSC (*P* = 0.003) and BM (*P* = 0.003). Grade II–IV aGVHD (*P* = 0.002) and chronic GVHD (*P* = 0.003) frequency decreased with CB when compared to allele matched PSC; however, aGVHD ratios did not change when we compared the CB patients with 8/8 HLA matched BM recipients, but chronic GVHD frequency decreased in the CB group (*P* = 0.01).

Basic factors associated with the success of the HSCT with CB are cell number and the degree HLA match. The New York Blood Center (NYBC) has analyzed 910 CB transplantations and revealed that products with ≥ 5 × 10^7^/kg cell count provided significantly higher 3-year survival rate [22]. The same data confirmed an absolute 3-year survival advantage of 25% with 6/6 HLA matched compared to 5/6 HLA matched CB HSCT. The joint CIBMTR/NYBC trial retrospectively evaluated 619 acute leukemia patients under age 16 during 1995–2003 period; 5-year survival ratios were higher with 6/6 HLA matched CB compared to 8/8 HLA matched BM transplantation (%63–%45). When there is one antigen mismatched CB, it is reasonable to increase the cell number (>3 × 10^7^/kg) with double donor to provide the same 5-year survival rate as HLA matched BM (%45) [23].

## 3. Conclusion

When we choose PSC instead of BM as the stem cell source, the following points should be beer in mind:

- chronic GvHD frequency increases,
- in advanced stage leukemias TRM decreases,
- in early phase CML cases TRM increases,
- in advanced stage CML patients survival rate increases,
- survival in chronic phase CML cases decreases,
- no effect can be achieved on relapse ratios,
- no difference on disease-free survival and overall survival on acute leukemia,
- aGVHD risk is similar with BM recipients in pediatric and adolescent acute leukemia patients, but chronic GVHD frequency is higher. Relapse ratios are similar in both BM and PSC groups. However, PSC increases TRM and overall mortality,
- aGVHD frequency is similar in aplastic anemia patients. Mortality increases in the group of patients under the age of 20,
- acute and chronic GVHD frequency increases with unrelated transplantations. Survival rates are similar to BM recipients.

When we use CB for HSCT, the following points should be emphasized:

- An HSCT candidate, but who does not have HLA matched sister/brother and who can provide adequate cord blood for transplantation, can be a recipient.
- The optimal graft selection procedure is still a matter of debate. The most important parameters are the number of nucleated cells and HLA match.
- The success of the cord blood practice depends on the primary disease, conditioning regimen, defrosting the product and the experience of the HSCT center.
- Especially when HLA mismatched unrelated and 5/6 HLA matched CB grafts are compared, CB can be a good alternative to unrelated transplantations.

## Figures and Tables

**Table 1:** Comparison of the stem cell sources.

	Cord Blood	PB	BM
Risk for the donor	None	Yes	Yes

Duration of searching (month)	≤1	3–6	3–6

Factors limiting the engraftment	Cell count	HLA match	HLA match

Dominant factor affecting the outcome	Engraftment failure delayed immune recovery	GVHD	GVHD

Minimal HLA match	4/6	9/10	9/10

Risk for GVHD	Low	High	High
Acute	Low	High	High
Chronic	Low	Higher	High

DLI possibility	None	Possible	Possible

Posttransplant infection risk	Higher	High	High

Immunotheraphy possibility	None	Yes	Yes
